# Drosophila TIEG Is a Modulator of Different Signalling Pathways Involved in Wing Patterning and Cell Proliferation

**DOI:** 10.1371/journal.pone.0018418

**Published:** 2011-04-08

**Authors:** Isabel Rodriguez

**Affiliations:** Centro de Biología Molecular Severo Ochoa CSIC-UAM, Universidad Autónoma de Madrid, Cantoblanco, Madrid, Spain; University of Texas MD Anderson Cancer Center, United States of America

## Abstract

Acquisition of a final shape and size during organ development requires a regulated program of growth and patterning controlled by a complex genetic network of signalling molecules that must be coordinated to provide positional information to each cell within the corresponding organ or tissue. The mechanism by which all these signals are coordinated to yield a final response is not well understood. Here, I have characterized the *Drosophila* ortholog of the human TGF-β Inducible Early Gene 1 (dTIEG). TIEG are zinc-finger proteins that belong to the Krüppel-like factor (KLF) family and were initially identified in human osteoblasts and pancreatic tumor cells for the ability to enhance TGF-β response. Using the developing wing of *Drosophila* as “in vivo” model, the dTIEG function has been studied in the control of cell proliferation and patterning. These results show that dTIEG can modulate Dpp signalling. Furthermore, dTIEG also regulates the activity of JAK/STAT pathway suggesting a conserved role of TIEG proteins as positive regulators of TGF-β signalling and as mediators of the crosstalk between signalling pathways acting in a same cellular context.

## Introduction

During the development of multicellular organisms, one of the main challenges is to understand how different signalling pathways that instruct cells to give rise to an organ with a characteristic size and shape are coordinated. Such growth and patterning programs are controlled by a set of evolutionary conserved signalling cascades.

Among them, TGF-β signalling stands out because of its ability to regulate diverse cellular processes including cell differentiation, cell proliferation, apoptosis and cell migration by means of the activation of specific genes in each developmental context [1]. Mutations in diverse components of the TGF-β transduction cascade are responsible for tumorigenesis and heritable disorders in humans [2].

*Drosophila* has provided many insights about the TGF-β signalling components and their molecular mechanisms [3], [4]. The imaginal wing disc is considered an ideal model system to study the role of TGF-β molecules in patterning and cell proliferation. In Drosophila there are seven TGF-β proteins, two activins (Activin-β, Daw) and three BMPs (Dpp, Gbb, Scw) acting through two different signalling cascades that include components either specific for each one (Babo, Smad2, Mad) or shared by both (Tkv, Pnt, Med) [5]. Phenotypic analysis suggests that both pathways are required for cell proliferation but only BMP pathway participates in patterning or cell differentiation. One of the best studied *Drosophila* BMPs is Decapentaplegic (Dpp), the ortholog of BMP2 [6]. Dpp acts as a long-range morphogen essential for patterning and growth of the wing disc [3]. Signalling propagation is initiated by the binding of Dpp ligand to the typeI/typeII receptor complex formed by *thick vein* (*tkv*) and *punt* (*pnt*) and the subsequent phosphorylation of Mad/R-Smad (P-Mad) in the cytoplasm. When P-Mad binds to Medea/Smad4, the P-Mad/Med complex is transcriptional active and enters the nucleus to activate target genes such as *spalt* (*sal*) [7] and *optomotorblind* (*omb*) [8] and to repress others like *brinker* (*brk*), a transcriptional repressor of Dpp target genes [9]. Brk represses Dpp signalling allowing the activation of *sal* and *omb* in the central region of the disc for the proper patterning of the wing. Other cofactors (Groucho, CtBP), extracellular proteins (Tld, Sog, Tsg, Cv, Cv-2) and repressors such as Schnurri and the I-Smad/Dad also contribute to shape Dpp activity revealing a more complex scenario around the tight regulation of this signalling pathway [3], [6].

The “TGF-β early response genes” (TIEG) proteins were first identified in human fetal osteoblasts as transcription factors induced by TGF-β signalling [10]. At the moment three TIEG proteins have been characterized: TIEG1 (KLF10), TIEG2 (KLF11) in humans and mice and TIEG3 in mice [10]–[12]. TIEG proteins belong to the broad family of Krüppel-like transcription factors (KLFs) (reviewed in [13]). They have three highly conserved zinc finger motifs and three repression (R1–R3) domains at the C- and N-terminus respectively [12], [14]. TIEG factors are evolutionary conserved from insect to vertebrates [15]. TIEG proteins can function as either activators [16]–[18] or repressors [19]–[21] by the direct binding to the gene promoter through specific GC-rich sequences. TIEG1, TIEG2 and TIEG3 enhance TGF-β/Smad signalling although their mechanisms are not identical [21], [22]. TIEG1 can regulate TGF-β/Smad signalling by induction of Smad2 expression and the repression of Smad7 [16], [19]. In addition, TIEG proteins participate in multiple developmental processes (osteoblasts, myoblasts, leukocytes, pancreatic beta-cells, etc) by the regulation of specific genes that control cell differentiation, cell proliferation and apoptosis [18],[23]–[25]. Moreover, TIEG1 acts as a mediator between different pathways acting in the same developmental context where TGF-β signalling is required [23], [26], It has been also observed that there is an inverse correlation between the level of TIEG1 and several type of cancer [23].

The present study shows that the *Drosophila* ortholog of TIEG1 protein (dTIEG) regulates growth and patterning of the wing acting as a positive modulator of both Dpp/BMP2 and JAK/STAT signalling. Furthermore, the control of JAK/STAT activity is not Dpp-dependent suggesting a conserved mechanism in which dTIEG plays a pivotal role to interconnect different signalling pathways.

## Results

### *cabut* gene encodes the *Drosophila* ortholog of TIEG proteins

**Figure 1:**
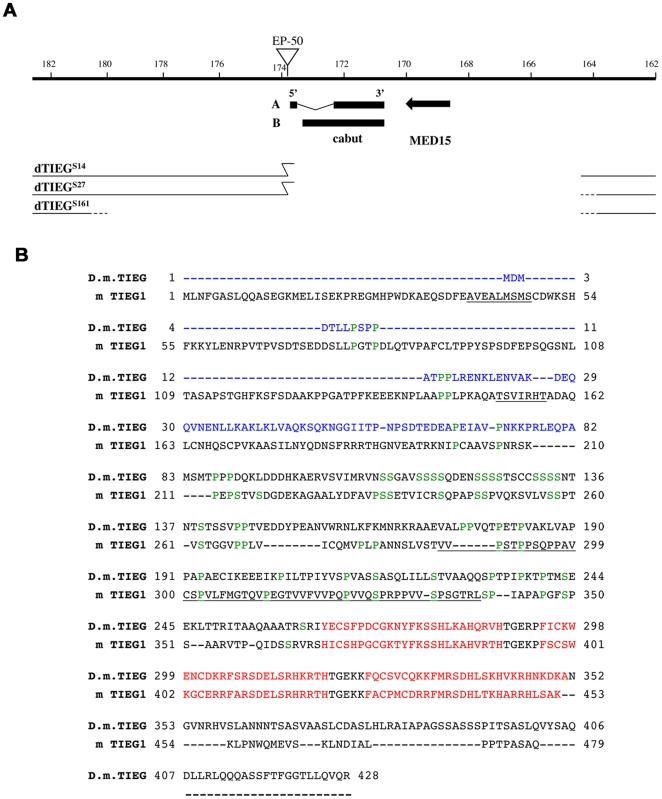
Molecular map of the dTIEG alleles in cabut locus. (A) Genomic region of the *cabut* locus showing the insertion of the original EP line (S50). The three novel *dTIEG* ^S14^, *dTIEG* ^S27^ and *dTIEG^S161^* alleles are deletions that completely eliminate *cbt* and the adjacent *MED15* gene. (B) Alignment of the amino acid sequences of dTIEG and mouse TIEG1 proteins. The amino acids (1–81) in blue are only present in the largest predicted dTIEG polypeptide. The three Zinc-finger motifs are highlighted in red. Scattered serine- and a proline-rich domains found in vertebrate TIEG proteins were shown in green. The predicted repressor domains (R1, R2 and R3) are underlined.

**Figure 2:**
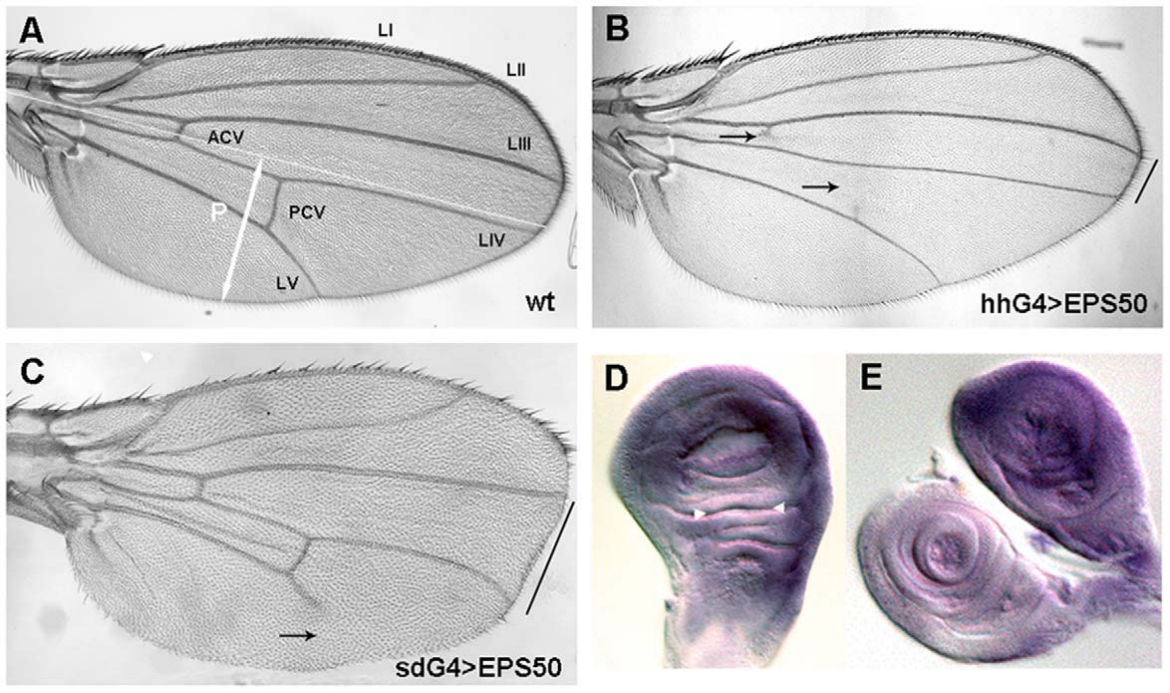
Overexpression of EPS50 causes alterations in the *Drosophila* adult wing. (A) wild-type wing and phenotypes displayed by *EPS50*; *hh-Gal4* (B) and *sd-Gal4*; *EPS50* (C). (B) Overexpression of EPS50 in the P compartment leads to partial loss of the crossveins (black arrows) and reduces the LIII-LIV intervein region. (C) Generalized expression increases the wing size, eliminates the distal LV vein (arrow) and almost entirely the D/V border cells. The five longitudinal wing veins (LI to LV) and the crossveins (ACV and PCV) are indicated in A. The white line delimits the A/P compartment border. Black lines show the intervein distance. (D, E) Expression pattern of dTIEG mRNA by in situ hybridization. The different levels of dTIEG expression are illustrated in (D) wing and (E) leg discs. Imaginal discs are the precursors of the adult cuticular structures. The white arrowheads in D point at the dorsal hinge of the wing disc.

In an overexpression screen to search for novel genes that contribute to the *Drosophila* wing pattern and growth EPS50 line was identified (see material and methods). This line was inserted in the 5′ UTR of the *cabut* (*cbt*) gene (http://flybase.bio.indiana.edu/) (Fig. 1A). Overexpression of EPS50 under the control of different Gal4 drivers causes growth and patterning defects such as an expansion of the intervein regions, loss of distal veins and notches in the D/V wing margin (Fig. 2). The phenotypes of the original EP line were reproduced when the largest cDNA was expressed under the same Gal4 drivers (not shown). *cbt* encodes two polypeptides of 428 and 346 aminoacids respectively. The predicted Cbt proteins differ in 82 aminoacids and show a strong similarity to members of the KLF superfamily [27]. A more detailed sequence analysis confirms that both proteins also contain the serine- and proline-rich regions between the N- and C-terminus only found in TIEG proteins and associated to the transcriptional repression domain R3 although the R1 and R2 domains seem to be incomplete (Fig. 1B). In the TGF-β pathway, TIEG proteins may act through a dual mechanism: increasing the levels of Smad2 [16] and repressing the inhibitory Smad7 [19], [21]. To carry out the genetic analysis during wing development new alleles were generated (see below) since the two reported *cbt* alleles, *cbt^EP2237E1^* and *cbt^EP2237E28^*, do complement with the deficiencies *BSC16* and *BSC107* that uncover the chromosomal region of the *cabut* locus (Table 1; [28]). The three new alleles were generated by imprecise excision of an isogenic line obtained from EPS50 insertion. They failed to complement each other and with the *Df(2L)BSC16* that uncovers this chromosomal region (Table 1; http://flybase.bio.indiana.edu/). Sequence analysis indicated that they consist of small deletions that uncover the *cbt* gene and the adjacent *MED15* gene separated by only 261 nucleotides and therefore they can be considered null *dTIEG* alleles (Fig. 1A). Thus, hereafter, the *cbt* gene will be named *Drosophila TIEG* (*dTIEG*) and the new alleles *dTIEG* ^S14^, *dTIEG* ^S27^ and *dTIEG^S161^* (Fig. 1A).

**Table 1:** Genetic complementation analysis of *cabut* alleles.

	dTIEG^S14^	cbt^EP(2)2237E1^	cbt^EP(2)2237E28^	PBac[WH]MED15^f04180^	ush^2^
Df(2L) BSC107/CyO (21C5-21D1)	−	+	+	−	*
Df(2L) BSC16/CyO (21C3-21C6-8)	−	+	+	**−**	−
Df(2L) Exel 8003 (21D1-21D2)	+	+	+	ND	+
ush^2^	+	+	+	ND	*
PBac[WH]MED15^f04180^	−	+	+	*	+
dTIEG^S14^	*	+	+	−	+

Abreviations: **Df** Chromosomal Deficiency, **+** mutations do complement, **−** mutations fail to complement, **ND** not determined.

In brackets are indicated the chromosomal interval.

### Altered expression of dTIEG causes growth and patterning defects in the wing disc by modulating Dpp signalling

**Figure 3:**
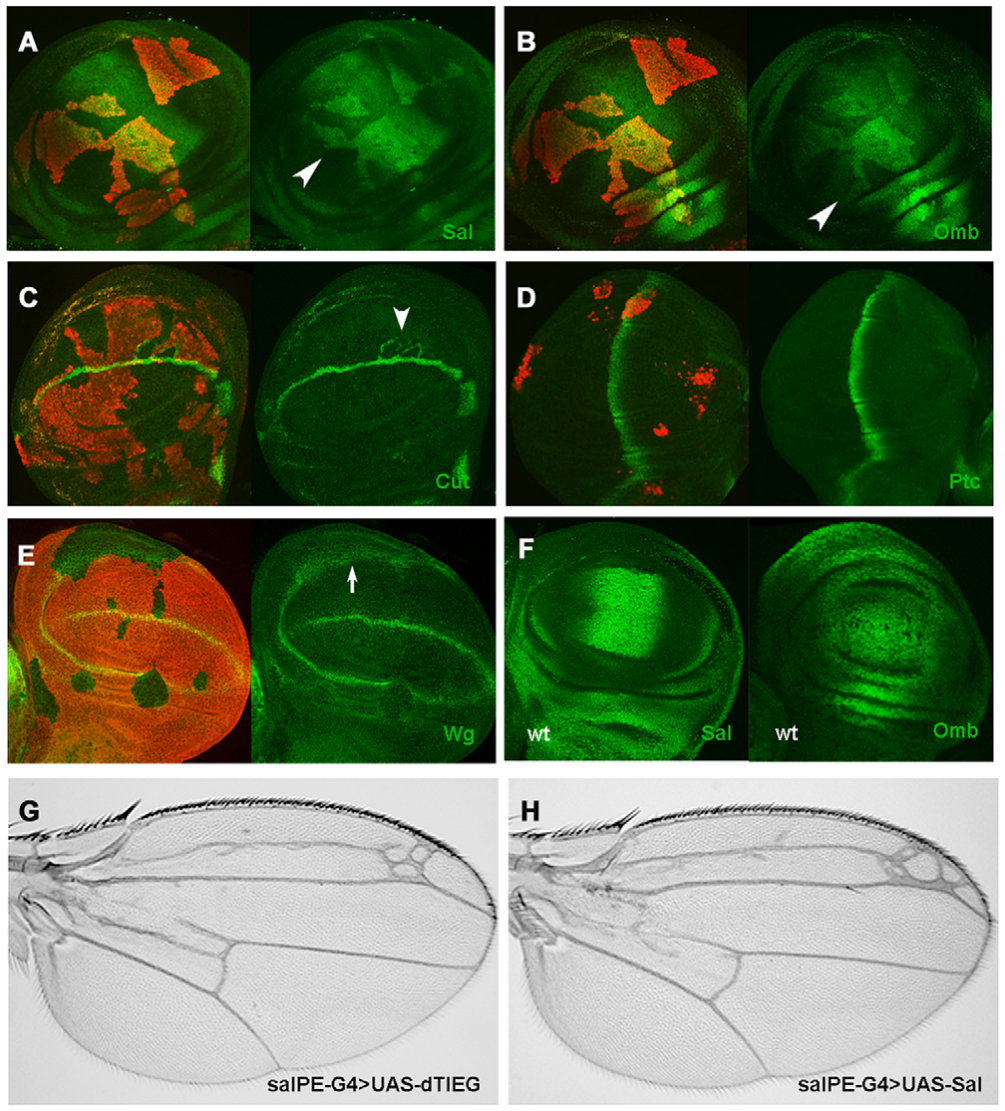
*dTIEG* expression regulates Dpp signalling. (A–D) Imaginal wing discs containing *UAS-dTIEG* clones marked in red. The Dpp target genes (A) Sal and (B) Omb are upregulated and ectopically expressed (arrowheads). (C) Cut (Ct) is ectopically expressed in some wild-type cells adjacent to the dTIEG-expressing clones in the central wing region but not within the clone, whereas (D) Patched (Ptc) expression is unaffected. Ct and Ptc are target genes of the Wg/Wnt and Hh pathways respectively. E) Distribution of Wg protein in *dTIEG^S14^/Minute* clones (absence of red marker) is more diffuse compared to wild-type cells probably as a consequence of the miss-regulation of Dpp/BMP2 signalling (F) Sal and Omb expression in wild-type wing discs. (G,H) Wing phenotypes displayed in flies expressing *UAS-dTIEG* and *UAS-Sal* under the *sal^PEv^-Gal4* driver. This driver is expressed in the central domain of Sal (F). Wings showed an altered size and severe defects in the vein pattern. The longitudinal LII and LIII veins are merged by extra vein material (compare to Fig. 2A).

Since both patterning and growth were altered in EPS50 wings and TIEG proteins are known to participate in TGF-β signalling, the involvement of dTIEG in Dpp/BMP2 signalling was next addressed. First, the dTIEG mRNA distribution was examined by in situ hybridization. In all the imaginal discs, dTIEG expression is quite generalized although not uniform (Fig. 2D,E). For instance, in the wing disc the mRNA levels in the dorsal hinge are less abundant than in the rest of the disc (Fig. 2D, white arrowheads). The observed phenotypes resemble defects found when pathways such Dpp/BMP2, Wingless/Wnt and Hedgehog (Hh) are altered. Therefore, *dTIEG* was overexpressed in clones and the expression of target genes of these pathways was analyzed in the wing disc. Whereas a strong upregulation of *sal* and *omb* expressions (two Dpp/BMP2 target genes) was observed in cells expressing *UAS-dTIEG* (Fig. 3A,B), no detectable difference was observed in the expression of Cut (Ct) and Patched (Ptc), target genes of the Wingless/Wnt and Hh pathways respectively (Fig. 3C,D). Occasionally, ectopic Cut expression was observed in wild-type cells adjacent to dTIEG expressing-cells (Fig. 3C arrowhead) probably due to an indirect effect on Wingless (Wg) diffusion (Fig. 3E arrow). Consistent with the observed Sal upregulation, ectopic expression of *UAS-dTIEG* in the central region of the wing using the *sal^EPv^*-*Gal4* driver (Fig. 3F) caused similar patterning phenotypes to those observed when *UAS-sal* was expressed under the same driver (Fig. 3G,H). Moreover, the wing size was also altered compared to a wild-type wing (compare to Fig. 2A).

**Figure 4:**
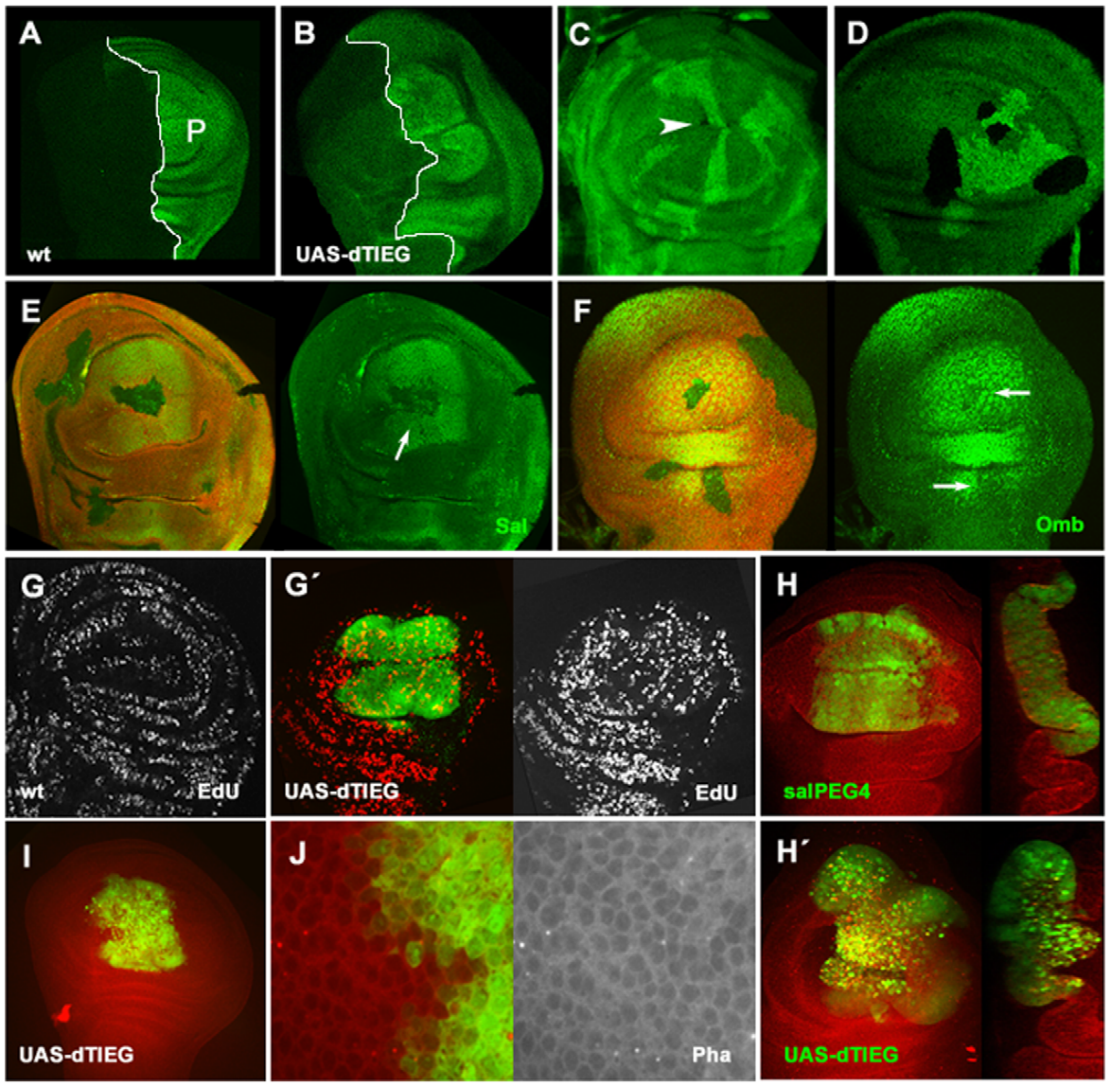
*dTIEG* regulates cell proliferation and modulates the expression of the *sal* and *omb* genes. Imaginal wing discs (A) wild-type and (B) *UAS-dTIEG/hh-Gal4* showing the posterior (P) cells marked in green by anti-En antibody. Overexpression of dTIEG causes overproliferation. Note the extra folds in the P compartment compared to A. A/P boundary is indicated with a white line. (C, D) *dTIEG* mutant clones induced by mitotic recombination at 24–48 h AEL (early) (C) and at 60 h AEL (late) (D) marked by the absence of GFP (green). In early *dTIEG^S14^* clones, mutant cells (black) do not survive compared to their sibling wild-type cells used as control (bright green). However, in *dTIEG^S14^* clones induced late the presence of mutant cells is increased but there are still fewer cells than in the control clone. (E, F) In a *dTIEG^S14^/Minute* genetic background, some *dTIEG* mutant cells survive even in early-induced clones. The loss of dTIEG function (absence of red marker) leads to a decrease of (E) Sal and (F) Omb expression (both in green). (G–H′) *UAS-dTIEG/sal^PEv^-Gal4(GFP)* imaginal discs. (G,G′) Incorporation of EdU in wild-type cells was uniform (G); in contrast, in dTIEG expressing-cells (green), EdU levels were increased (green). (I,J) Rhodamine-labeled phalloidin staining (grey) was used to examine the cell size in *UAS-dTIEG/sal^PEv^-Gal4(GFP)* wing discs. A high magnification of (I) show similar size and shape in wild-type and *UAS-dTIEG* cells (green). (H,H′) A strong overexpression of dTIEG causes epithelial disorganization probably due to massive cell death (bright spots).

For a detailed analysis of dTIEG contribution in cell proliferation the effect of *UAS-dTIEG expression* was studied in the wing disc using two different drivers: *hh-Gal4* in the P compartment and *sal^EPv^*-*Gal4* in the central region of the pouch. In these conditions, the wing discs showed a P compartment and wing pouch region (Fig. 4B,H′ green) considerably bigger than wild-type wing discs (Fig. 4A,H green). To determine whether the enlarged domains were due to an increase in the cells numbers, EdU incorporation was examined (Fig. 4G,G′ grey). dTIEG cell-expressing domain showed a higher number of EdU positive cells. On the contrary, the cell size was unaffected by dTIEG over-expression as indicated by rhodamine-labeled phalloidin staining, suggesting that the enlarged territories reflect an increase in cell numbers rather than cell size (Fig. 4I,J grey). Contribution of a decrease in apoptosis to these phenotypes was ruled out because in wing discs this is a rare phenomenon [29].

These results suggest that *dTIEG* might control both patterning and cell proliferation via the regulation of the Dpp/BMP2 signalling.

Next, *dTIEG* expression was eliminated in somatic loss-of-function clones using the FRT/FLP method and analyzed in the wing imaginal disc (Xu and Rubin, 1993). In *dTIEG^S14^* clones induced early (24–48 hours AEL (after eggs laying)) the survival of the mutant cells (black) was drastically reduced (Fig. 4C). When the *dTIEG^S14^* clones were induced later (>60 hours AEL) mutant cells were recovered although clones were smaller than their sister clones (bright green) and showed smooth borders (Fig. 4D). At this developmental time, in most of the induced *dTIEG^S14^* clones the expression of Dpp/BMP2 target genes was nearly unaffected (not shown). To further explore the requirements of dTIEG function, the *Minute* technique was used to provide a proliferative advantage to mutant cells [30]. In this genetic background, dTIEG mutant cells were recovered in the wing disc when clones were induced early. The expression of Sal and Omb in *dTIEG^S14^/Minute* clones was cell-autonomously reduced although differences in the expression level were observed ranging from a severe decrease to sporadically complete absence (Fig. 4E,F green channel; Fig. S1). Strikingly, in *dTIEG^S14^/Minute* clones induced later (72 hours AEL) Sal and Omb expression was nearly unaffected in most of the cases (not shown) suggesting that dTIEG function is required for Dpp/BMP2 signalling modulation only at early stages of wing development.

Taken together, these results indicate that dTIEG can regulate cell proliferation and patterning during wing development. Moreover, the described alterations are caused by the modulation of Dpp/BMP2 signalling by dTIEG as indicated by the changes observed in the expression of Dpp target genes *sal* and *omb*.

### Analysis of *MED15* function in wing development

**Figure 5:**
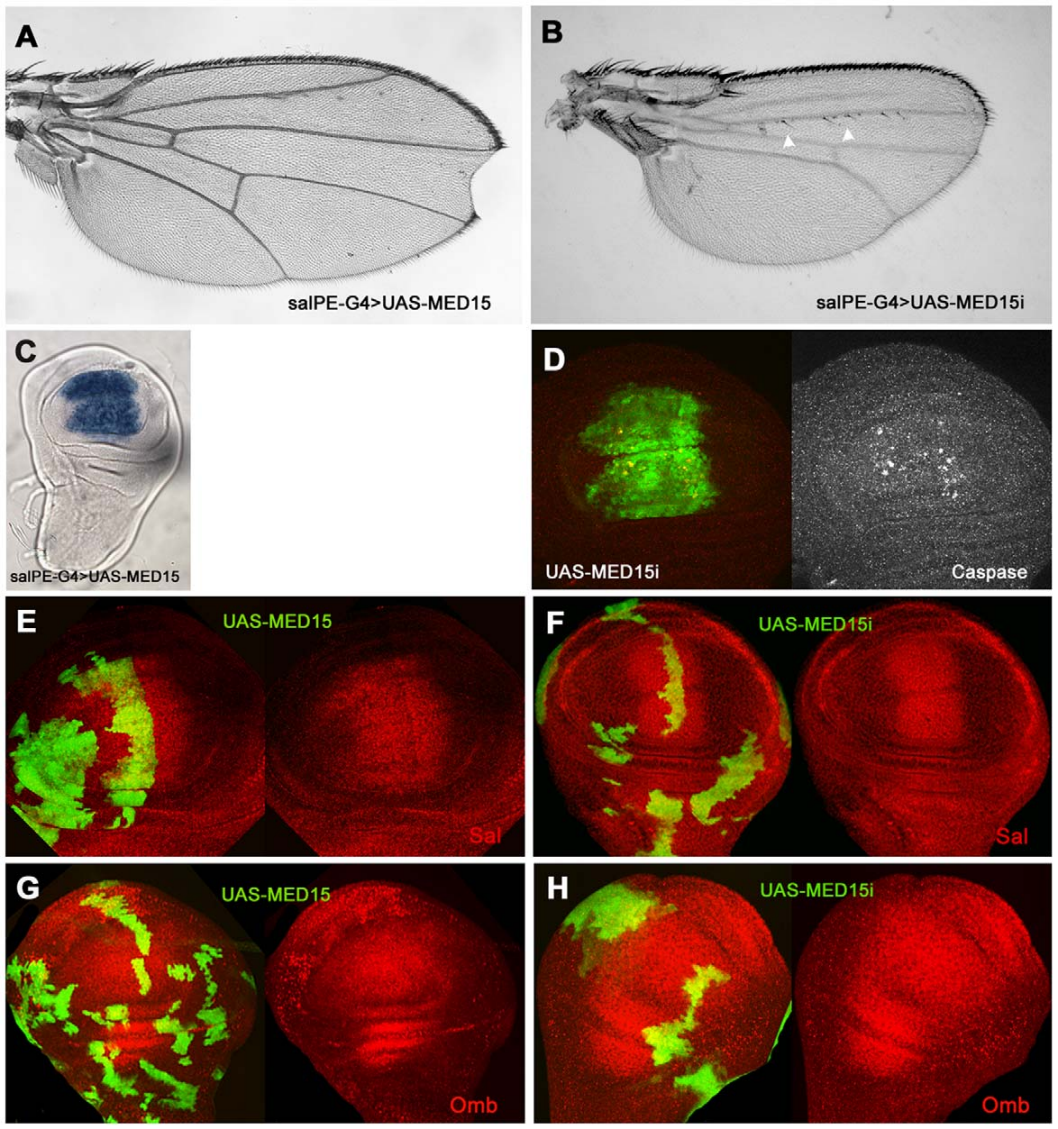
Wing phenotypes caused by miss-expression of *MED15*. Representative wings expressing either (A) *UAS-MED15* or (B) *UAS-MED15i* under the *sal^PEv^-Gal4* driver. MED15 overexpression does not cause patterning and growth defects except for a notch in the D/V border that is occasionally observed. In contrast, the *UAS-MED15i* wing is much smaller in size and shows an absence of the distal part of the LII vein and the appearance of ectopic sensory organs along LII (arrowheads). This could be a consequence of the reduction in the wing size. (C) Expression of *MED15* mRNA in a *UAS-MED15/sal^PEv^-Gal4* wing disc. (D) *MED15* RNAi induces cell death. *UAS-MED15i/sal^PEv^-Gal4(GFP)* cells showed expression of activated Caspase3 (grey). (E–F) Wing imaginal discs expressing either *UAS-MED15* or *UAS-MED15i* in clones (green). Neither the Sal (C, D) nor Omb (E, F) expression levels are modified compared to those observed in wild-type cells.

The above observations point out directly to a role of *dTIEG* in Dpp/BMP2 signalling similar to the vertebrate TIEG proteins in TGF-β signalling; however given that the molecular lesion of *dTIEG* alleles also eliminates the adjacent *MED15* gene, a contribution of this gene to the described phenotypes cannot be ruled out. MED15 encodes a small protein that is a component of the Mediator complex [28]. This complex acts as an adapter to recruit transcription factors to the basal transcriptional machinery and regulate the tight control of gene expression [31]. To further analyze the contribution of MED15 function during wing development, adult wing phenotypes were examined when MED15 function was either increased (*UAS-MED15*) or decreased by the expression of RNA interference (*UAS-MED15i*) under the control of *sal^PEv^-Gal4* (Fig. 5). Most of the *UAS-MED15* wings did not display any patterning or size defects compared to the wild-type wing while a small percentage showed a notch in the wing margin (compare Fig. 5A and 2A). Whereas, in *UAS-MED15i* wings the vein patterning is unaffected the wing size is significantly reduced and reproduces the reported phenotypes for *med15* alelles (Fig. 5B) [28]. According to this study, cell death was increased in *UAS-MED15i* expressing-cells in the wing disc (Fig. 5D). In the same experimental conditions, the expression levels of Sal and Omb analyzed in *UAS-MED15* and *UAS-MED15i* expressing-clones were similar to those of wild-type cells (Fig.5E–H). Mutant clones of *med15*, using a strong hypomorph allele, induced (48–72 hours AEL) in the wing disc using are viable and with normal clone borders [28]. A slight reduction of Sal expression was observed when the clones were located in the lateral border of Sal domain. Similarly, Bs expression, a target of Hh signalling during vein formation, was also decreased [28].

Since overexpression of MED15 did not resemble the wing phenotypes of *UAS-dTIEG* and *med15* loss of function only affects the basal activity of different signalling pathways, the wing patterning and groth defects of the novel *dTIEG* alleles described above can be assigned to dTIEG function. However, a contribution of MED15 to the low cell survival of the *dTIEG^S14^* cells cannot be ruled out.

### *dTIEG* function is Mad-dependent in Dpp/BMP2 signalling

**Figure 6:**
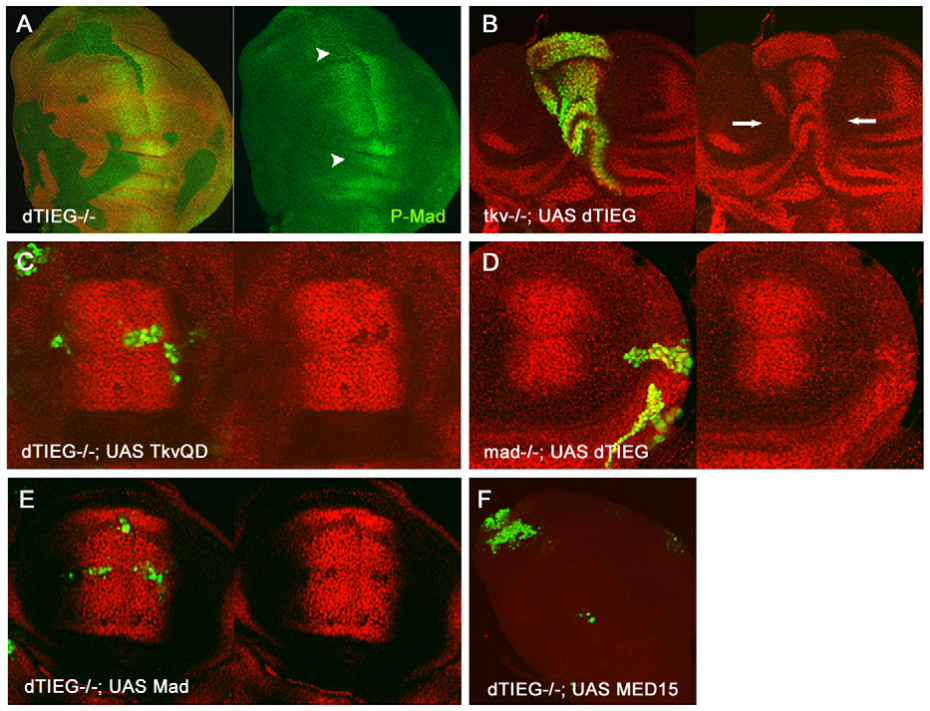
dTIEG requires P-Mad for the activation of Dpp/BMP2 target genes. (A) The expression of P-Mad is slightly reduced in some *dTIEG^S14^Minute* cells (absence of red marker) (arrowheads). (B-E) Sal expression domain in the central region of wing discs (red channel). (B) In *tkv* mutant cells (green) that ectopically express *dTIEG*, Sal expression is restored even in the absence of Tkv and the increased cell proliferation deforms the wing territory and causes extra folds (arrows). (C) In *dTIEG^S14^* clones expressing Tkv^QD^, the mutant cells are unable to upregulate Sal expression due to the absence of dTIEG. Tkv^QD^ is a constitutive active form of the Dpp receptor Tkv. (D) *mad^12^* clones expressing dTIEG do not survive in the wing pouch (see Material and Methods). Moreover, in a *mad^12^;UAS-dTIEG* clone located laterally the overexpression of dTIEG cannot upregulate Sal expression as observed in wild-type cells (compare to Fig. 3A). (E) Similar to (C), the expression of Mad in *dTIEG^S14^* cells is unable to upregulate Sal expression as occurs in wild-type cells. In all panels, clone cells are marked by the expression of GFP (green) and stained with the Sal antibody (red). (F) MED15 expression cannot rescue the cell viability of *dTIEG* mutant cells. From all the *dTIEG;UAS-MED15* wing discs analyzed (n = 60) only one showed cells expressing GFP (presence of *dTIEG^−/−^*; *UAS-MED15* cells) indicating that increased levels of MED15 reduce the cell viability. The illustrated clones in the wing discs are representative examples in size and position of the clones recovered for each genotype.

To understand the mechanism by which *dTIEG* modulates Dpp/BMP2 signalling, the expression of Mad/R-Smad was also analyzed in *dTIEG^S14^/Minute* clones [32]. Similar to what it was observed for Sal and Omb, P-Mad expression is decreased but not completely eliminated in these clones (Fig. 6A, arrowheads in green channel; Fig. S1).

To gain more insights into dTIEG function a mosaic analysis with a repressible cell marker (MARCM) was also performed [33] using Sal expression to monitor the activity of the Dpp/BMP2 pathway [34]. By this technique, the function of specific genes is eliminated while simultaneously other genes are ectopically expressed within the clone. It must be emphasized that the recovered *tkv^a12^*, *dTIEG^S14^*and *mad^12^* clones have a small size or do not survive due to their low cell viability (not shown).

First, *tkv^a12^* clones that ectopically expressed dTIEG were analyzed. While in *tkv^a12^* cells the expression of Sal is absent [35], upon ectopic expression of dTIEG, Sal expression is recovered at wild-type levels (Fig. 6B, red). Moreover, the size of *tkv^a12^*; *UAS-dTIEG* clone indicates that the low cell viability of *tkv^a12^* cells is now recovered when dTIEG is expressed. Conversely, the expression of an activated form of Tkv (Tkv^QD^) in *dTIEG^S14^* clones could not rescue the loss of Sal expression or cell viability of the *dTIEG* mutant cells (Fig. 6C red). Moreover, the strong Sal upregulation and overgrowth caused by Tkv^QD^ expression in wild-type cells was compensated by elimination of dTIEG function [32]. These observations suggest that *dTIEG* acts downstream of the Tkv receptor.

Next *UAS-dTIEG* was expressed in *mad^12^* cells. Whereas ectopic expression of *UAS-dTIEG* in wild-type cells causes Sal upregulation (Fig. 3A), in *mad^12^*; *UAS-dTIEG* cells Sal expression could not be restored (Fig. 6D, arrowhead in red channel). Furthermore, no *mad^12^* clone could be recovered at the central region of the wing disc (n = 30) suggesting that the dTIEG expression was unable to rescue the reduced cell viability of *mad^12^* cells. Similarly, ectopic *UAS-Mad* in *dTIEG^S14^* clones did not restore endogenous Sal expression (Fig. 6E, red) or produce overproliferation as in wild-type cells [36]. This epistatic relationship between *mad* and *dTIEG* suggests that dTIEG might act either downstream of or in parallel to Mad. Furthermore, *dTIEG^S14^* clones expressing *UAS-MED15* could not be recovered in wing discs (n = 60) indicating that ectopic MED15 expression reduces even more the cell viability (Fig. 6F). It should be noted that this was also observed in wild-type cells (Fig. 5A).

Taken together, these results led to conclude that dTIEG acts on Dpp/BMP2 pathway downstream of *tkv* and requires Mad to exert its function not only in the activation of the Dpp targets but also in the transduction of Dpp signal to control cell survival and proliferation.

### The repressor *brinker* is not upregulated in *dTIEG^S14^* cells with reduced Dpp activity

In vertebrates, “in vitro” experiments have demonstrated that TIEG proteins can modulate TGF-β signalling by a dual mechanism: increasing the levels of a transcriptional activator as Smad2 [16] and repressing the inhibitory Smad7 [14]. In the wing disc, the repressor of Dpp target genes is *brinker* (*brk*) [37]. Brinker is expressed at lateral regions of the wing where Dpp/BMP2 activity does not occur (Fig. S1). In the central region the activation of P-Mad expression by Dpp/BMP2 signalling represses *brk* transcription to yield a nested pattern of Sal and Omb [3]. Conversely, the ectopic expression of Brk acts negatively in *sal* and *omb* expression.

**Figure 7:**
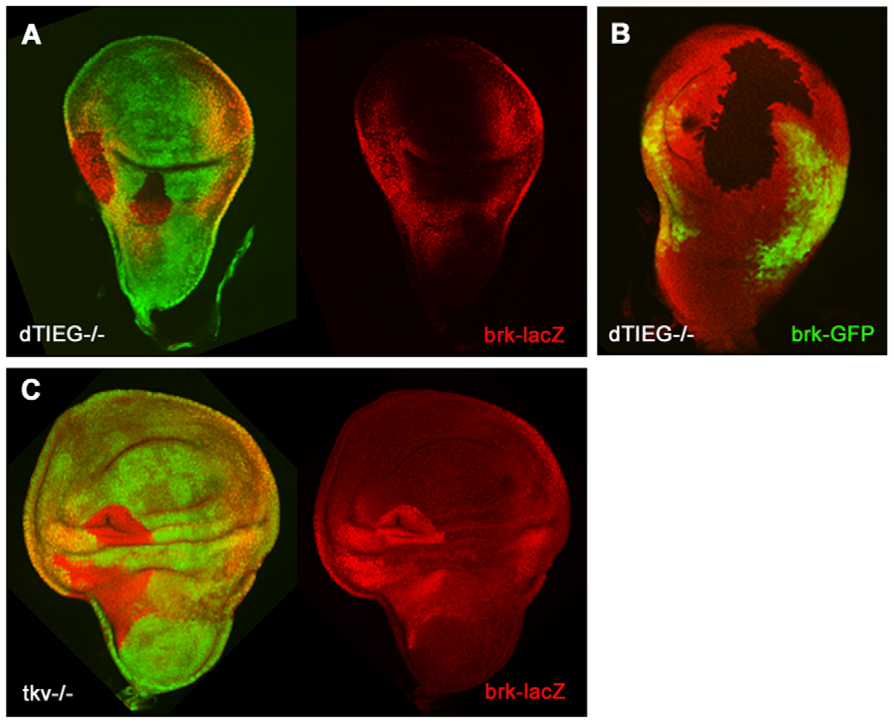
*brk* expression is not regulated by dTIEG. *brk* expression was monitored by either *brk-lacZ* (A, C) or *brkGal4>UASGFP* (B). (A) Elimination of *dTIEG* function in *dTIEG^S14^*/Minute clones does not produce detectable changes in brk expression in lateral regions of the wing disc or (B) upregulation of *brk* expression in the central region, even though Sal, Omb and P-Mad levels are decreased (compare to Fig. 4E,F and 6A). In contrast, (C) when Dpp/BMP2 signalling is eliminated as in *tkv^a12^* clones, *brk-lacZ* is upregulated at any position.

Since *brk* downregulation requires P-Mad expression and P-Mad levels are reduced in *dTIEG^S14^* cells, it was investigate whether dTIEG might also regulate the expression of *brk* repressor. To test this possibility, expression of *brk* was examined in *dTIEG^S14^/Minute* clones and compared to *tkv^a12^/Minute* clones. In *dTIEG^S14^* clones located at lateral positions of the disc *brk* expression was unaffected (Fig. 7A, red). In agreement with this, neither Sal nor Omb were ectopically expressed in *dTIEG* mutant cells (Fig. 4E,F). Moreover, in *dTIEG* clones at the central region of the wing disc *brk* expression was undetectable (n = 17, Fig. 7B) despite the fact that Sal expression was decreased or eliminated (Fig. 4E). On the contrary, in *tkv^a12^/Minute* clones, where Dpp/BMP2 activity is depleted, the expression of *brk* was upregulated at any position of the wing disc (Fig. 7C). These data suggest a different requirement of P-Mad for the activation of Dpp target genes and the repression of *brk*, since in dTIEG mutant cells the reduced P-Mad levels are still sufficient to repress *brk* in the wing pouch.

### *dTIEG* controls the JAK-STAT signalling pathway

Cell proliferation in the wing disc responds to a complex genetic program in which other signalling pathways, in addition to Dpp/BMP2, are known to contribute. The Dpp/BMP2 mechanism to promote uniform cell proliferation from a gradient of Dpp is not well understood. It has been proposed the existence of unknown regulators that might allow an integrated action of other pathways to give rise to the final uniform proliferation [38]. The results presented here indicate that the modulation of Dpp/BMP2 signalling by dTIEG seems to be critical for cell proliferation while other pathways, such as Hh and Wg, seems to be unaffected by dTIEG. Another important pathway that controls patterning and cell proliferation in the *Drosophila* imaginal disc is JAK/STAT [39]. Previous studies have shown that there is an interaction between JAK/STAT and other signalling pathways such as Wg, Dpp and Notch during development. In the wing disc, mutations of this pathway lead to a decrease in cell proliferation [40].

**Figure 8:**
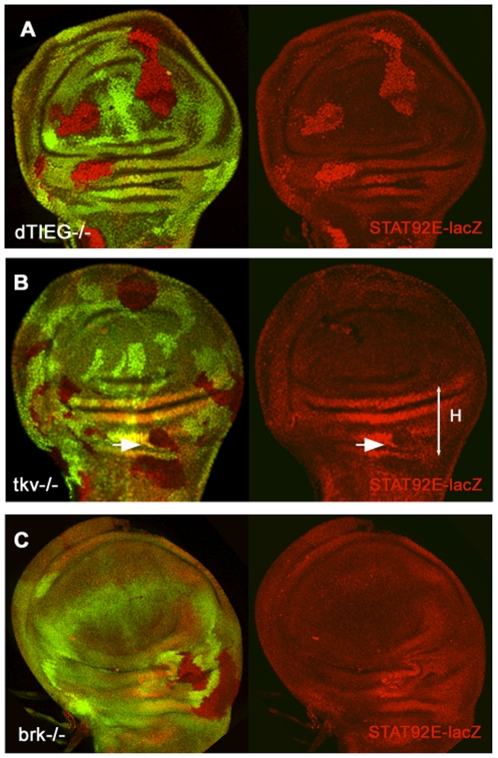
*dTIEG* regulates the activity of the JAK/STAT pathway. The *STAT92E-lacZ* expression (red), used as a reporter of JAK/STAT activity, is preferentially observed in the hinge (H) region. (A) In *dTIEG^S14^*cells (absence of GFP) the *STAT92E-lacZ* expression is upregulated at any position of the wing pouch. On the contrary, *STAT92E-lacZ* expression is not altered when the activity of Dpp/BMP2 pathway is either (B) eliminated as in *tkv^a12^* cells or (C) increased as in *brk^M68^* cells. In fact, in some *tkv^a12^* clones located in the hinge region (H) *STAT92E-lacZ* expression is downregulated (arrow).

To analyze whether dTIEG could be regulating JAK/STAT signalling, the *STAT92E-lacZ* reporter was monitored in *dTIEG^S14^* clones. *STAT92E-lacZ* is an enhancer trap insertion into the gene that encodes the *Drosophila* STAT protein [41]. The expression pattern of *STAT92E-lacZ* is complementary to Dpp/BMP2 signalling and is confined to the proximal wing showing higher levels in the dorsal hinge (Fig. S1). Published data indicate that high levels of *STAT92E-lacZ* reflect a decreased activity of the pathway [42]. In *dTIEG^S14^* clones *STAT92E-lacZ* expression is upregulated (Fig. 8A, red) and, in agreement with the reported data, this could be associated to the low rate of cell proliferation observed in *dTIEG^S14^* cells. To test whether Dpp/BMP2 signalling was involved, *STAT92E-lacZ* expression was analyzed in *tkv^a12^* and *brk^M68^* clones and in both genetic backgrounds the expression of *STAT92E-lacZ* was not affected (Fig. 8B,C). These data indicate that *dTIEG* can regulate JAK/STAT activity independently of its function on Dpp/BMP2 pathway, since neither an upregulation (*brk*) nor a downregulation (*tkv*) of Dpp signalling cause the same effect on *STAT92E-lacZ* expression.

## Discussion

Here, it has been studied the function of dTIEG, the *Drosophila* ortholog of TIEG1 protein, during the imaginal discs development. Similar to TIEG1 protein in humans, the dTIEG expression in the imaginal discs is ubiquitous although the transcriptional levels vary [23]. dTIEG shares structural features with the vertebrate dTIEG proteins such as the three Zn-finger motifs and a serine- proline-rich region, where the R3 repression domain would be located [10], [15]. However, the R1 and R2 motifs are more divergent suggesting that these domains might not be completely conserved and therefore the repressor function of dTIEG could be compromised.

Another important difference with respect to TIEG proteins is that dTIEG enhances BMP signalling, particularly the Dpp signalling pathway [16]. The genetic analysis has provided evidence that dTIEG is a novel regulator of patterning and growth during wing development modulating positively both the Dpp and JAK/STAT pathways. When dTIEG and Sal are overexpressed, the wing phenotypes are similar. dTIEG controls Dpp/BMP2 signalling by modulating the expression of P-Mad and the target genes Sal and Omb. In *Drosophila*, there are two more BMP ligands; Scw that is required only in early embriogenesis [43] and Gbb that contributes to BMP signalling with moderate effects in late patterning and cell proliferation during wing development [44]. Similarly, the Activin pathway also functions during wing development although its role is less understood. Two different ligands dAct and Daw trigger signalling through the type I receptor Baboon and Smad2, both specific components of this pathway, to regulate cell proliferation and in a lesser extent patterning [45], [46]. Recent data indicate that Smad2 exerts an inhibitory effect on Mad signalling that suggest a role of Smad2 on vein formation and cell proliferation through Dpp/BMP2 signalling [47]. Thus, according to the phenotypes described here the regulation of these pathways by dTIEG can be ruled out. Other KLF members identified in *Drosophila* such as Krüppel, Sp1 and Buttonhead are involved in developmental processes independent of Dpp/BMP2 signalling [48], [49].

### dTIEG is a positive regulator of the Dpp pathway

Previous results had shown that Cabut is expressed in the embryo and regulates *dpp* expression acting downstream of the JNK pathway during dorsal closure [27]. dTIEG modulates Dpp/BMP2 signalling during wing development. Several pieces of evidence support this conclusion. First, dTIEG overexpression enhances transcriptional activation of Dpp target genes such as *sal* and *omb* as it is the case with the overexpression of an active form of the TGF-β type II receptor Tkv [32]. Target genes of other signalling pathways, such as Hedgehog or Wingless, do not seem to be directly affected [14]. In contrast, the elimination of dTIEG function in somatic clones causes a down-regulation of *sal* and *omb* expression indicating a decrease of Dpp/BMP2 activity in the wing disc. Moreover, P-Mad expression is also reduced. Besides, the epistatic experiments revealed that dTIEG acts downstream of Tkv and requires Mad as a partner to exert its regulatory action on *sal* and *omb* genes. However, a slight decrease of dTIEG function caused by two independent lines of targeted expression of interference RNAs (*UAS-dTIEGi*) did not cause any discernible phenotype (Fig. S2). These results indicate that dTIEG must be completely eliminated to exert its regulatory function on Dpp/BMP2 pathway and further reinforce the role of dTIEG as a modulator in contrast to other components of the pathway that have been shown to induce severe phenotypes when eliminated.

Since the function of dTIEG on the Dpp/BMP2 pathway is reminiscent of the role of TIEG proteins in TGF-β signalling, the expression of Dpp/BMP2 repressors was also examined. The overexpression of TIEG1 and TIEG3 results in the repression of the inhibitory Smad7 [16], [21]. In *Drosophila*, however, the elimination of dTIEG function did not cause detectable changes in the expression of either the I-Smad/Dad (data not shown) or Brk suggesting certain differences in the mechanism of action of dTIEG. These observations could be explained by the absent of two repressor domains (R1 and R2) in dTIEG. Moreover, recent studies in mouse myoblasts have showed that TIEG1 can be stimulated by both pathways: myostatin and TGF-β signalling [50]. In this context the expression of Smad2 and Smad7 was unaffected in contrast to the changes observed when TGF-β signalling was activated [16], [19]. This suggests that myostatin signalling might compensate the TGF-β signalling on the regulation of Smad2 and Smad7. In *Drosophila*, the Myoglianin (Myg) is another TGF-β ligand related to Myostatin. “In vitro” experiments indicate that Myg can trigger activin signalling through Wit, another TGF-β type II receptor, that binds both activin and BMP ligands through a mechanism that is poorly understood [51]. These results indicate that many aspects about the mechanism of TIEG proteins still remain unknown and suggest that TIEG might be using alternative mechanisms in different cellular contexts.

### dTIEG regulates cell proliferation

Misregulation of the Dpp pathway not only leads to alterations in patterning but also in cell proliferation. Whereas mutant cells (i.e *tkv* clones) that cannot respond to the Dpp/BMP2 signal fail to proliferate and die, an increase of Dpp signalling promotes overproliferation [35]. Previous studies have postulated different models to correlate the uniform cell growth in the wing disc with the slope of the Dpp gradient [52] and *brk* activity [53], [38]. The existence of a still unknown inhibitor of cell proliferation has been suggested [3]. However, other signalling pathways also contribute to wing proliferation and the integration of all these inputs must be considered although the mechanism by which the net balance arises remains unclear.

The above results demonstrate that dTIEG controls cell proliferation. Ectopic dTIEG expression promotes overproliferation whereas elimination of dTIEG function in cell clones using a null allele produces a failure in cell proliferation. To assess that the loss of function phenotypes were caused by dTIEG and not for the adjacent *med15* gene a genetic analysis of *med15* was performed in the wing disc. The results are consistent with a role of MED15 as a co-activator required for the basal transcription of different genes that results essential for cell viability.

On the other hand, dTIEG also regulates the expression of STAT92E, the main effector of the JAK/STAT pathway. The upregulation of *STAT92E-lacZ* expression in *dTIEG* mutant cells reflects a decrease in JAK/STAT activity indicating that dTIEG is also a positive regulator of this pathway. The result fits with the reduced size of *dTIEG* mutant clones respect to the sibling clones (wild-type cells) and the proliferative effect described for STAT92E in the wing disc [40]. Thus, the JAK/STAT pathway might contribute to the defects in cell proliferation observed in dTIEG cells. Several pieces of evidence support a role for JAK/STAT in the regulation of other signalling pathways although in most of the cases the mechanism remains unknown. In other *Drosophila* developmental contexts, STAT92E can upregulate *dpp* signalling [54] and repress the Wingless and Hh pathways [55]. Thus, *dTIEG* could play a role as a connector gene to integrate signalling from Dpp/TGF-β and JAK/STAT pathways. Indeed, the mild reduction of P-Mad levels observed in *dTIEG* mutant cells could reflect the net balance resulting from simultaneous changes in the JAK/STAT and Dpp/BMP2 activities. Supporting this observation, TIEG1, in addition to its role in the transcriptional control of Smad proteins, also regulates the activity of other genes by binding directly to their promoters [26].

In conclusion, our results demonstrate an evolutionary conserved function of TIEG proteins regulating the activity of different TGF-β signals and mediating the crosstalk among different pathways in the control of differentiation and cell proliferation. Further experiments will be required for the acquisition of a better knowledge of the molecular mechanism involved in the process.

## Materials and Methods

### Drosophila Strains

Mutant alleles and transgenes for *brk*, *mad*, *tkv*, *med15* and *Df(2L)BSC16* and *BSC107* are described in Flybase (http://flybase.bio.indiana.edu/). The molecular lesions of the three novel *dTIEG* alleles were characterized by PCR using primers to the P element ends and the flanking genomic DNA region. The EPS50 line was isolated in a overexpression screen (I.Guerrero and G.Carrillo unpublished). The *UAS-dTIEG* and *UAS-MED15* transgenic flies were made from the cDNAs SD05726 and GH03922 respectively. The *UAS-MED15i* ((NIG-Fly 4184R-4)) and two lines of *UAS-dTIEGi* (NIG-Fly 4427R-1 and VDRC 5044) that express MED15 RNAi and dTIEG RNAi respectively were obtained from the stocks centers: NIG-Fly (http://www.nig.ac.jp/) and VDRC (http://stockcenter.vdrc.at/control/main).

### Generation of somatic clones

Loss-of-function clones were generated by FLP/FRT and MARCM techniques ([56]; [33]). The following chromosomes were used:

*y w hs-Flp*; *FRT40A dTIEG^S14^*, *y w hs-Flp*; *FRT40A tkv^a12^*, *y w hs-Flp*; *FRT40A mad^12^*, *y w brk^M68^ f^ 36^ FRT18A*; *FRT40A ubi-GFP*, *FRT40A tub-Gal80*; *STAT92E-lacZ*, *FRT40A tub-Gal80*; *UAS-dTIEG*, *FRT40A tub-Gal80*; *UAS-MED15*, *FRT40A tub-Gal80*; *UAS-Mad* and *FRT40A tub-Gal80*; *UAS-Tkv^QD^*. To verify that the low rate of recovered clones in the MARCM experiments was not due to the experimental conditions control clones were induced in parallel using *FRT40A ubi-GFP* to monitor the appearance of twin spots in the wing disc. Larvae were heat shocked for 1 hour at 37°C and left to develop at 25–29°C. *UAS-dTIEG*, *UAS-cbti*, *UAS-MED15* and *UAS-MED15i* were ectopically expressed using the following Gal4 drivers: *sd-Gal4*, *sal^EPv^*-Gal4 [57], *hh-*Gal4 and *Act>y+>Gal4*; *UAS-lacZ*. Second instar larvae were heat-shocked 10–15 min at 37°C and left to develop at 25–29°C.

### EdU labeling

For cell proliferation experiments, DNA synthesis was measured using EdU (5-ethynyl-2′-deoxyuridine) using the following protocol (by B. Perez-San Juan): Larvae were dissected in Schneider medium (SM) and incubated in SM+1% FCS containing 10 mM EdU (Invitrogene) for 15 minutes at room temperature. After 3 rinses in PBS, larvae were fixed for 1 hr in 4% paraformaldehyde. Then they were washed 3 times in PBT (PBS+0,1% Triton X-100). Detection of EdU was done by incubation in Click-iT reaction cocktail (+Alexa Fluor 555 Azide) for 30 minutes at room temperature (Invitrogene). After 3 washes in PBS/BSA3% and one more in PBT, imaginal discs were mounted in 70% glycerol (in PBS).

### Inmunohistochemistry and in situ hybridization

Imaginal discs were dissected and stained as described previously [58]. The following antibodies were used: mouse anti-Ptc (1∶100), mouse anti-Cut (1∶100) and mouse anti-En (1∶100) from Developmental Studies Hybridoma Bank; rabbit anti-Sal (1∶100 a gift from JF. De Celis), rabbit anti-P-Mad (1∶500 a gift from G. Morata), mouse anti-Omb (1∶400 a gift from G.O. Pflugfelder), rhodamine-labeled phalloidin (Sigma), rabbit anti-Caspase3 (Cell Signalling), rabbit anti-β-galactosidase (1∶10000 Cappel), and mouse anti-β-galactosidase (1∶500 Promega). Fluorescent secondary antibodies were from Jackson ImmunoResearch Laboratories. The imaginal discs were mounted in Citifluor fluorescent medium (Electron Microscopy Sciences). Wing discs and adult wings images were acquired using a Zeiss LSM510 Confocal Microscope (fluorescence samples) and a Zeiss Axiovert200 (bright-field) microscope respectively.

To analyze mRNA distribution, in situ hybridization was performed as described [59]. To prepare the antisense *dTIEG* RNA probes the full-length cDNA SD05726 (dTIEG) and a 560 pb (dTIEGi) fragment were used to detect endogenous mRNA in *UAS-dTIEGi* wing discs. dTIEGi sequence is located at the 3′ end of SD05726 cDNA and does not overlap with the targeted sequences used for RNA interference assays in *UAS-dTIEGi* wing discs (see Figure S2).

For the alignment of mouse TIEG1 and *Drosophila* Cabut proteins the EDL08798.1 and EDX03233.1 sequences were used.

## Supporting Information

Figure S1 **Expression pattern of different markers in wing disc and dTIEG mutant clones.** (A) Wild-type wing discs showing in green the expression pattern of the different target genes analyzed. (B,C) Early-induced *dTIEG^S14^Minute* clones in which the mutant territory (absence of red) is exceptionally large. These clones are infrequent. Note the decreased number of mutant cells that deform the wing discs. In these *dTIEG^S14^* clones Omb expression is completely absent and Sal expression is reduced in the central domain and eliminated in the lateral region.
(TIF)

Figure S2 ***dTIEG*** **mRNA expression and wing phenotype of** ***UAS-dTIEGi*** **.** (A) The nucleotide sequences of two independent RNAi constructs used to knockdown dTIEG expression are indicated in green and blue respectively within the dTIEG cDNA sequence. In purple are indicated the sequence used to generate an antisense dTIEG RNA probe to specifically detect endogenous mRNA expression when the dTIEG RNAi was expressed. (B) *dTIEG* mRNA expression in wild-type and *UAS-dTIEGi*/*hh-Gal4* wing discs. Note that the dTIEG mRNA levels in posterior P cells. are still quite high when both RNAi constructs were expressed either independently or in combination. (C) Wing of *UAS-dTIEGi*/*hh-Gal4* flies showed a minor effect on growth such as a slight reduction of the wing size compared to the wild-type wing (wt) or a weak patterning defect such as elimination of wing margin cells (black arrow). These results indicate that the *dTIEG* RNAi constructs are not too efficient in eliminating dTIEG function. (D) Apoptosis is activated in *UAS-dTIEG/sal^PEv^-Gal4(GFP)* cells visualized by Caspase3 expression (grey).
(TIF)
