# Supplementary figures and images for: Drosophila TIEG Is a Modulator of Different Signalling Pathways Involved in Wing Patterning and Cell Proliferation
Source: PLoS One. 2011 Apr 8;6(4):e18418. doi: 10.1371/journal.pone.0018418 (PMC3072976; doi:10.1371/journal.pone.0018418)

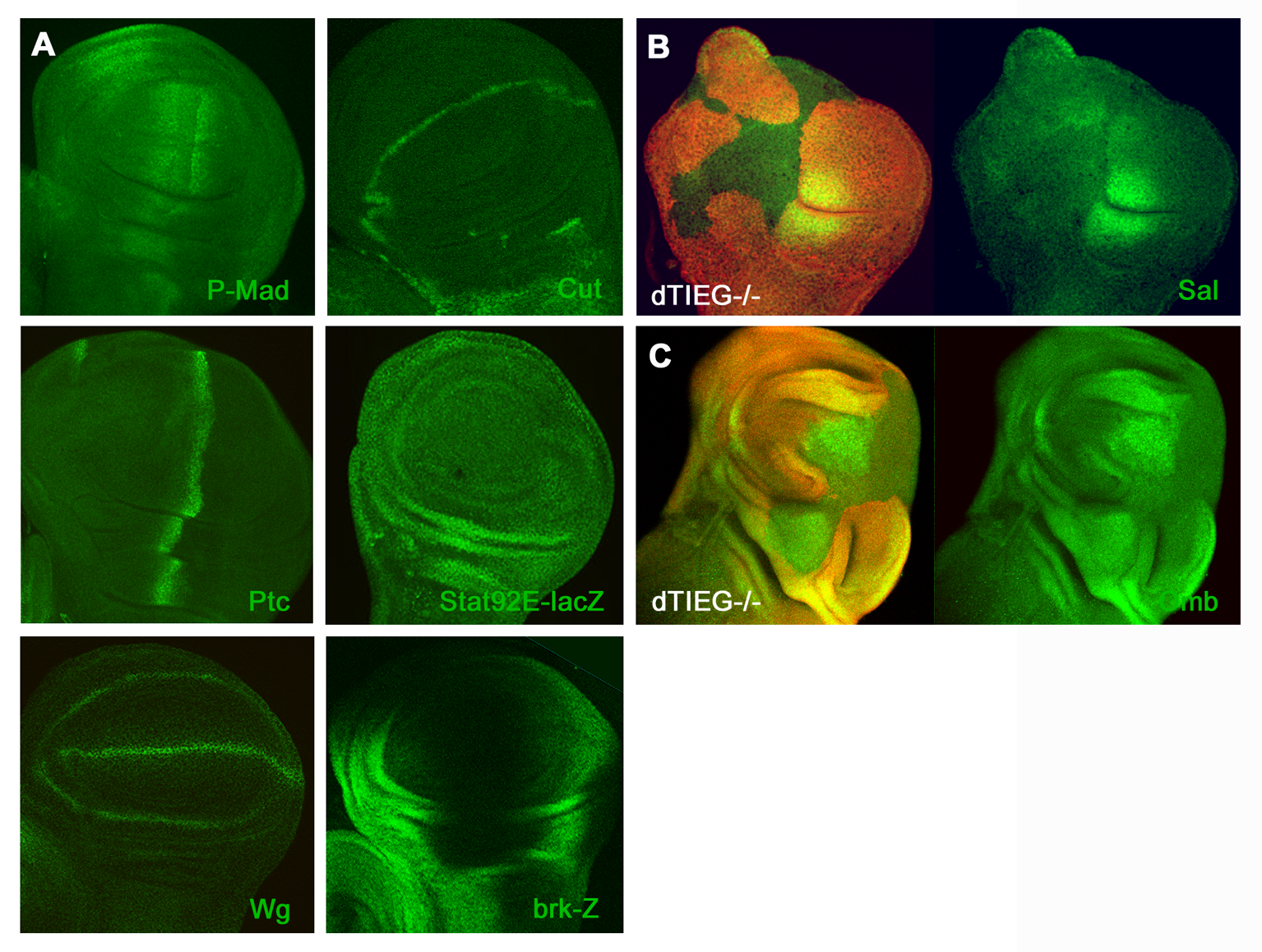

Supplement: Figure S1 — Expression pattern of different markers in wing disc and dTIEG mutant clones. (A) Wild-type wing discs showing in green the expression pattern of the different target genes analyzed. (B,C) Early-induced dTIEGS14Minute clones in which the mutant territory (absence of red) is exceptionally large. These clones are infrequent. Note the decreased number of mutant cells that deform the wing discs. In these dTIEGS14 clones Omb expression is completely absent and Sal expression is reduced in the central domain and eliminated in the lateral region. (TIF) [file pone.0018418.s001.tif]

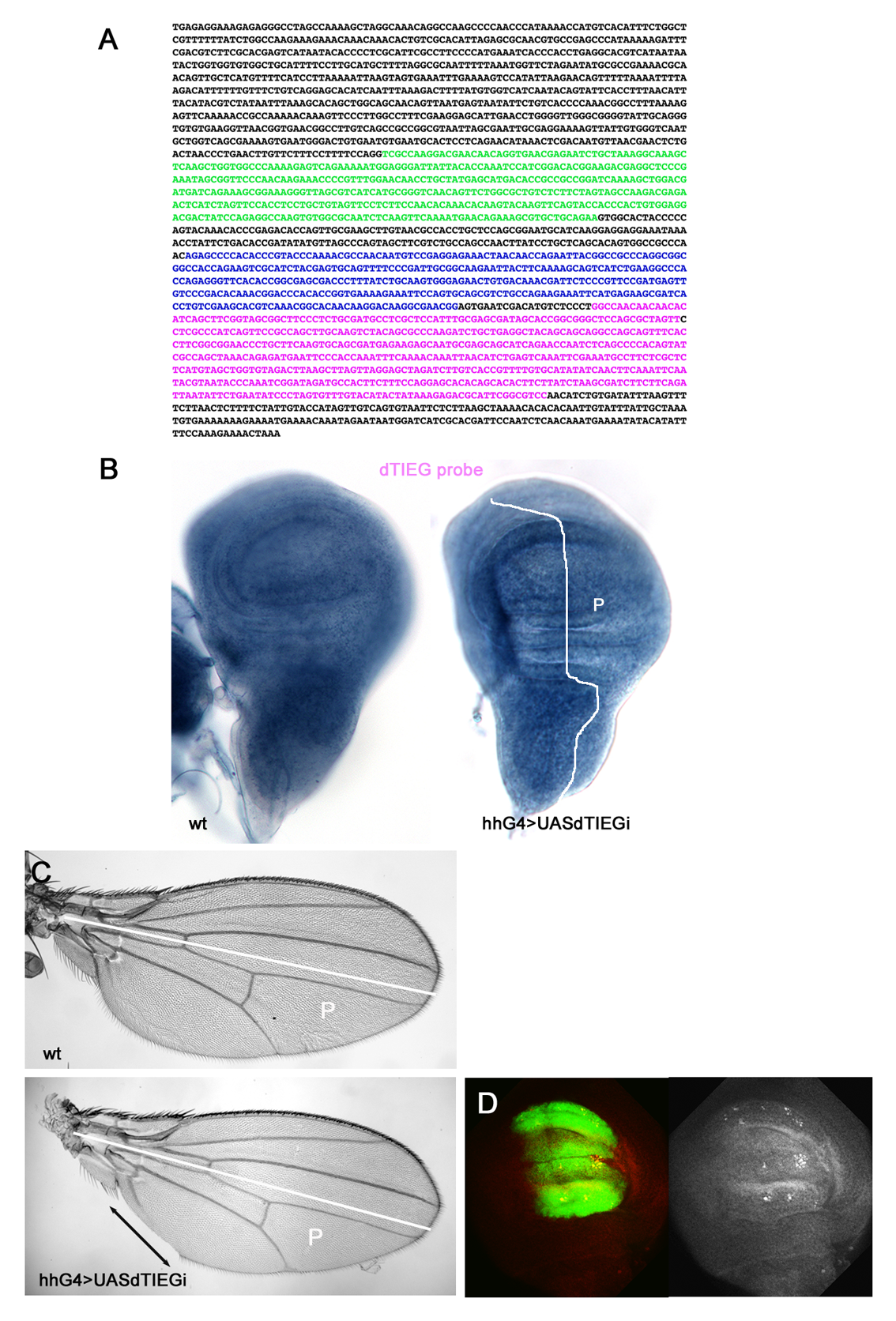

Supplement: Figure S2 — dTIEG mRNA expression and wing phenotype of UAS-dTIEGi . (A) The nucleotide sequences of two independent RNAi constructs used to knockdown dTIEG expression are indicated in green and blue respectively within the dTIEG cDNA sequence. In purple are indicated the sequence used to generate an antisense dTIEG RNA probe to specifically detect endogenous mRNA expression when the dTIEG RNAi was expressed. (B) dTIEG mRNA expression in wild-type and UAS-dTIEGi/hh-Gal4 wing discs. Note that the dTIEG mRNA levels in posterior P cells. are still quite high when both RNAi constructs were expressed either independently or in combination. (C) Wing of UAS-dTIEGi/hh-Gal4 flies showed a minor effect on growth such as a slight reduction of the wing size compared to the wild-type wing (wt) or a weak patterning defect such as elimination of wing margin cells (black arrow). These results indicate that the dTIEG RNAi constructs are not too efficient in eliminating dTIEG function. (D) Apoptosis is activated in UAS-dTIEG/salPEv-Gal4(GFP) cells visualized by Caspase3 expression (grey). (TIF) [file pone.0018418.s002.tif]
